# Supplementary material for: Genome-Wide Analysis of the Rab Gene Family in Melilotus albus Reveals Their Role in Salt Tolerance
Source: Int J Mol Sci. 2022 Dec 21;24(1):126. doi: 10.3390/ijms24010126 (PMC9820615; doi:10.3390/ijms24010126)
Supplement: Supplementary file 1 [file ijms-24-00126-s001.zip › Description of Supplementary Figures -C.Z.pdf]

Supplementary Figures

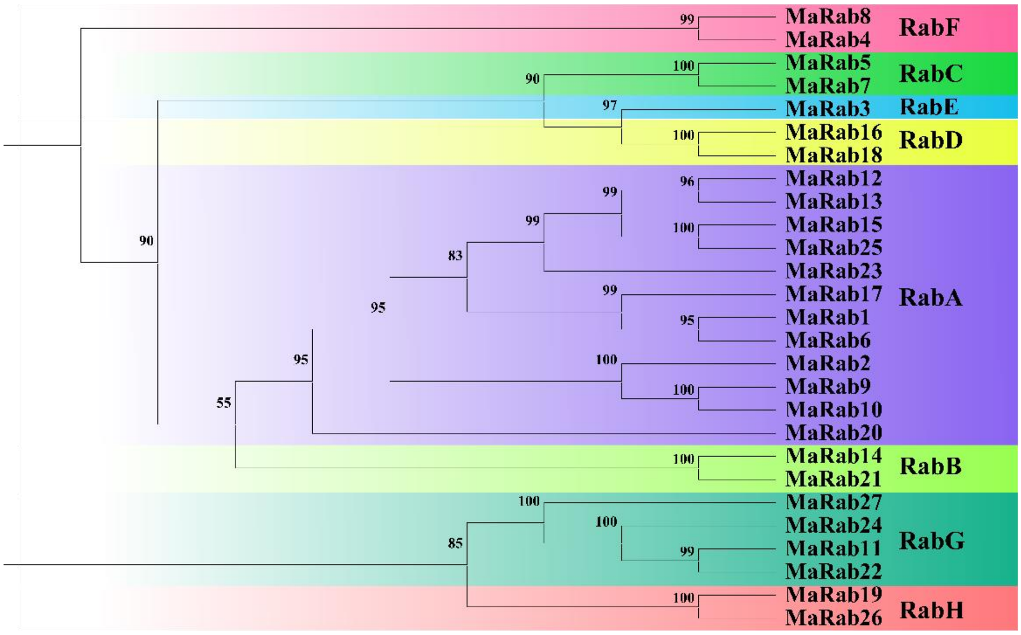

Figure S1. Phylogenetic tree of 27 identified *Rabs* from *M. albus* were contracted using the MEGA 7 program [63]. These members were clustered into 8 groups (A–H) and indicated by different colors.

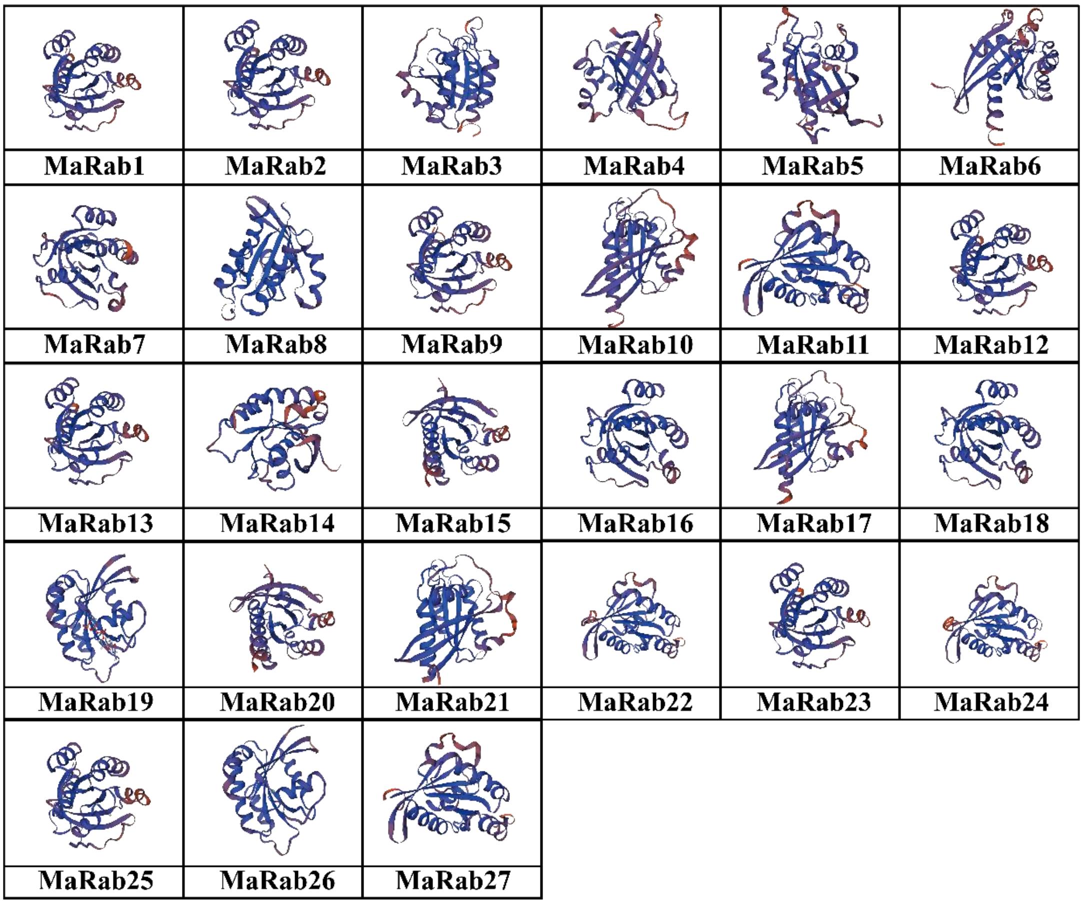

Figure S2. Protein 3D structure prediction of *Rab* proteins from *M. ablus*.

Notes: The figure shows the most likely 3D structure of each gene.

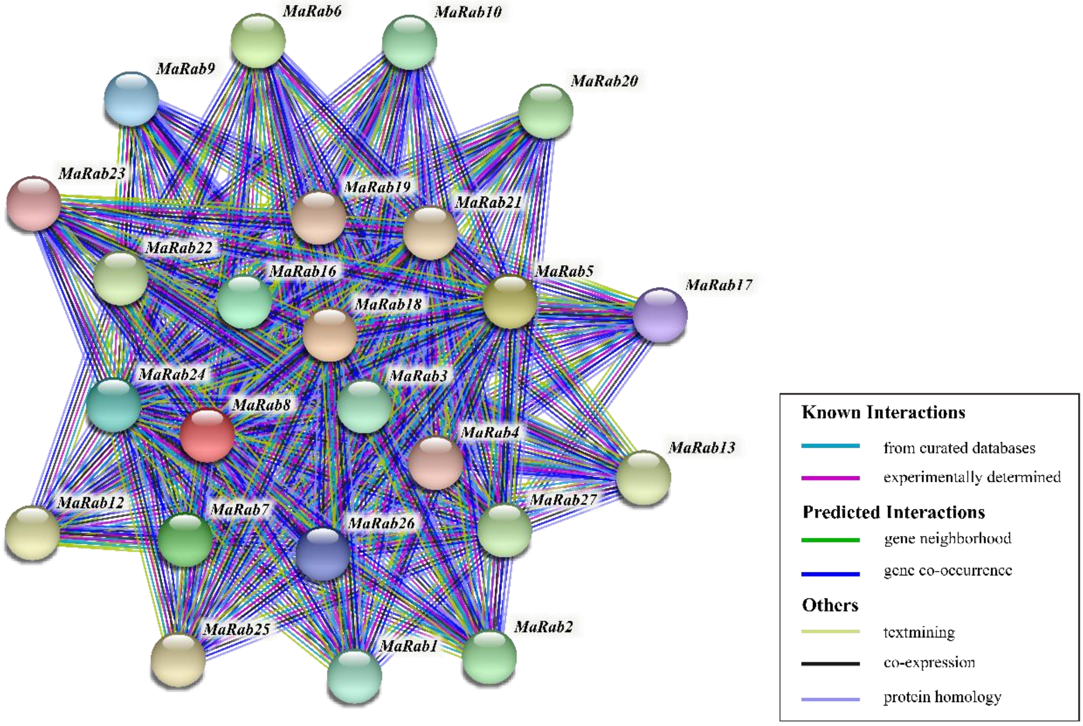

Figure S3. Protein network generated by STRING (v 11.5) for *MaRab* genes. Colored nodes represent proteins whereas the different colour of lines represents the types of evidence (depicted by the colour legend) for the association.

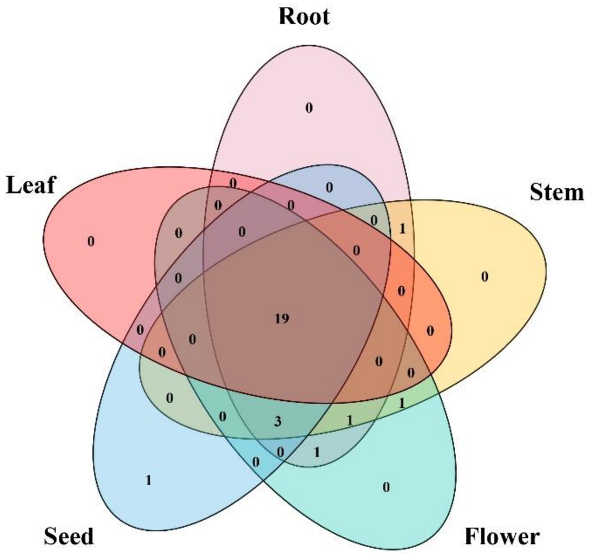

Figure S4. Venn diagram showing DEGs in five tissues (leaf, root, stem, flower and seed) of MaRab gene family in *M. ablus*.

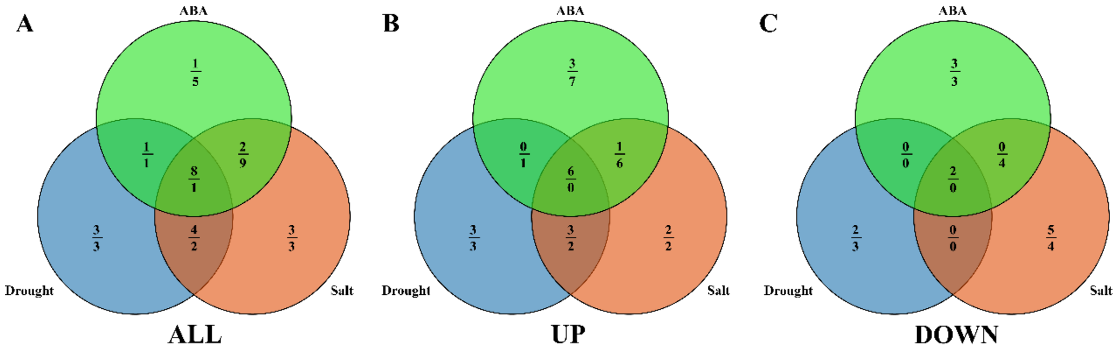

Figure S5. DEGs of *MaRab* gene family in *M. ablus* under salt, ABA and drought stress. (**A**–**C**) Venn diagram of (**A**) all, (**B**) upregulated and (**C**) downregulated DEGs between different treatment in shoot and root *.

* The number above the line represents the number of above-ground DEGs and the number below the line represents the number of root DEGs.
