# Supplementary material for: Genome-Wide Analysis of the Rab Gene Family in Melilotus albus Reveals Their Role in Salt Tolerance
Source: Int J Mol Sci. 2022 Dec 21;24(1):126. doi: 10.3390/ijms24010126 (PMC9820615; doi:10.3390/ijms24010126)
Supplement: Supplementary file 1 [file ijms-24-00126-s001.zip › Table S1.pdf]

Table S1. Analysis of amino acid sequence characteristics of *MaRab* gene family in *M. albus*.

Gene name	Gene ID	Localization*	Protein length (aa)	Molecular weight	Theoretical pI	Grand average of hydropathicity (GRAVY)
MaRab1	Malbus0100901.1	Cytoplasmic	223	24605	6.84	-0.242
MaRab2	Malbus0101242.1	Cytoplasmic	223	24909.11	5.38	-0.376
MaRab3	Malbus0102734.1	Extracellular	216	23899.97	6.61	-0.368
MaRab4	Malbus0102808.1	Cytoplasmic/Chloroplast	205	22256.22	5.24	-0.145
MaRab5	Malbus0103131.1	Extracellular/Chloroplast	211	23379.67	5.46	-0.246
MaRab6	Malbus0103708.1	Cytoplasmic/Mitochondrial	221	24570.04	6.17	-0.201
MaRab7	Malbus0105099.1	Cytoplasmic/Nuclear	211	23729.08	6.41	-0.316
MaRab8	Malbus0203104.1	Chloroplast	200	21884.84	6.75	-0.219
MaRab9	Malbus0203653.1	Cytoplasmic/Nuclear	219	24360.22	5.12	-0.342
MaRab10	Malbus0301562.1	Cytoplasmic/Chloroplast	215	23954.84	4.85	-0.319
MaRab11	Malbus0400281.1	Cytoplasmic	207	23249.31	5.4	-0.379
MaRab12	Malbus0400908.1	Cytoplasmic/Chloroplast	218	24082.11	5.72	-0.234
MaRab13	Malbus0401587.1	Cytoplasmic	217	24005.03	5.87	-0.273
MaRab14	Malbus0403058.1	Cytoplasmic/Mitochondrial	210	22961.06	6.52	-0.179
MaRab15	Malbus0404520.1	Cytoplasmic	217	24184.22	5.34	-0.271
MaRab16	Malbus0500112.1	Cytoplasmic/Golgi	203	22477.33	5.11	-0.292

MaRab17	Malbus0501082.1	Cytoplasmic	235	25872.39	5.17	-0.206
MaRab18	Malbus0503821.1	Golgi	202	22430.32	5.53	-0.331
MaRab19	Malbus0601485.1	Chloroplast	207	23034.29	7.67	-0.166
MaRab20	Malbus0603614.1	Cytoplasmic	216	24511.98	5.24	-0.325
MaRab21	Malbus0604274.1	Cytoplasmic/Mitochondrial	211	23254.35	6.9	-0.263
MaRab22	Malbus0700190.1	Cytoplasmic	207	23281.36	5.61	-0.386
MaRab23	Malbus0702319.1	Cytoplasmic/Mitochondrial	215	23698.69	6.22	-0.279
MaRab24	Malbus0702537.1	Cytoplasmic/Chloroplast	206	23071.9	5	-0.441
MaRab25	Malbus0703028.1	Cytoplasmic	217	24199.27	5.63	-0.249
MaRab26	Malbus0704571.1	Cytoplasmic/Chloroplast	207	23191.46	6.39	-0.248
MaRab27	Malbus0603614.1	Cytoplasmic	206	23236.44	6.16	-0.358

* Subcellular localization after the slash is predicted as the second most likely location.
