# Supplementary material for: Genome-Wide Analysis of the Rab Gene Family in Melilotus albus Reveals Their Role in Salt Tolerance
Source: Int J Mol Sci. 2022 Dec 21;24(1):126. doi: 10.3390/ijms24010126 (PMC9820615; doi:10.3390/ijms24010126)
Supplement: Supplementary file 1 [file ijms-24-00126-s001.zip › Table S3.pdf]

Table S3. Number of *MaRab* genes in each group according to introns amount.

Group \ No. of Introns	1	2	3	4	5	6	7	Total
A	12	0	0	0	0	0	0	12
B	0	0	0	0	2	0	0	2
C	0	0	0	0	2	0	0	2
D	0	0	0	0	0	0	2	2
E	0	0	0	0	0	0	1	1
F	0	0	0	0	0	1	1	2
G	0	0	0	0	0	4	0	4
H	0	0	0	0	2	0	0	2
Total	12	0	0	0	6	5	4	27
