# Supplementary material for: Genome-Wide Analysis of the Rab Gene Family in Melilotus albus Reveals Their Role in Salt Tolerance
Source: Int J Mol Sci. 2022 Dec 21;24(1):126. doi: 10.3390/ijms24010126 (PMC9820615; doi:10.3390/ijms24010126)
Supplement: Supplementary file 1 [file ijms-24-00126-s001.zip › Table S4.pdf]

Table S4. Cis-acting elements in promoters of *MaRab* genes.

Gene ID	auxin-responsive	cell cycle regulation	defense and stress responsiveness	gibberellin-responsiveness	light responsiveness	low-temperature responsiveness	salicylic acid responsiveness	abscisic acid responsiveness	anaerobic induction	auxin responsiveness (MYB binding site)	circadian control	light responsiveness (MYB binding site)	seed-specific regulation	MeJA-responsiveness	zein metabolism regulation	meristem expression	endosperm expression	anoxic specific inducibility	gibberellin-responsive (MYB binding site)	drought-inducibility	flavonoid biosynthetic genes regulation	light induction	MYBHv1 binding site	wound-responsive element	Total
MaRab1	0	0	0	0	0	3	2	1	2	0	0	1	0	4	0	1	0	0	0	1	0	0	0	0	15
MaRab2	0	0	1	0	0	1	0	2	0	0	0	3	0	0	0	0	0	0	0	1	0	0	0	0	8
MaRab3	0	0	0	0	0	0	1	0	1	0	0	0	0	2	0	0	1	0	0	0	0	1	0	0	6
MaRab4	0	0	0	0	0	1	1	0	0	0	0	0	0	2	0	1	0	0	0	0	0	0	0	0	5
MaRab5	1	0	0	0	0	1	0	4	2	1	0	6	0	0	0	1	0	0	1	0	0	0	0	0	17
MaRab6	1	1	0	0	0	0	1	2	2	0	0	1	0	0	0	0	1	0	0	0	0	0	2	0	11
MaRab7	1	0	2	0	0	3	1	1	5	1	0	3	0	10	3	0	0	1	0	1	0	1	1	0	34
MaRab8	1	0	0	0	1	2	0	8	2	0	0	11	0	4	0	0	0	0	0	0	0	0	0	0	29
MaRab9	1	0	0	0	1	0	0	1	5	0	0	2	0	6	0	0	1	0	0	0	0	0	0	0	17
MaRab10	0	0	1	0	0	0	0	3	2	0	0	2	0	0	0	1	1	0	2	0	0	1	0	1	14
MaRab11	0	0	1	0	1	0	1	1	0	0	0	2	0	2	0	0	0	0	0	0	0	0	1	0	9
MaRab12	1	0	1	0	0	1	0	3	4	0	0	3	0	0	1	1	0	0	0	0	0	0	0	0	15

MaRab13	0	0	0	0	0	1	1	0	2	0	0	0	0	6	0	0	0	0	1	0	0	0	0	0	11
MaRab14	0	0	1	0	0	0	0	0	1	0	1	1	0	0	0	0	0	0	0	0	0	0	0	0	4
MaRab15	0	0	1	0	0	1	1	0	3	0	1	0	0	4	1	0	0	0	0	0	0	2	0	0	14
MaRab16	0	0	1	1	0	1	0	2	2	0	1	2	0	4	0	1	0	0	0	0	0	0	3	0	18
MaRab17	0	0	0	0	0	0	1	1	5	0	1	0	0	4	0	1	1	0	0	1	0	0	2	0	17
MaRab18	2	0	0	0	0	0	0	8	0	0	0	8	0	12	0	0	0	0	0	0	0	0	0	0	30
MaRab19	1	0	0	1	0	0	0	2	2	0	1	2	1	2	0	0	1	0	0	2	1	1	0	0	17
MaRab20	0	0	3	0	2	3	1	1	3	0	0	1	0	4	4	0	0	0	0	0	0	0	0	0	22
MaRab21	2	0	1	0	1	1	0	2	1	0	1	2	0	4	0	3	0	0	2	0	0	0	0	0	20
MaRab22	0	0	0	0	0	0	2	0	0	1	0	0	0	0	0	1	2	0	0	0	0	0	0	0	6
MaRab23	0	0	0	0	1	0	0	0	0	0	0	1	0	4	1	0	0	0	0	0	0	0	1	0	8
MaRab24	1	0	2	0	0	2	0	0	1	0	1	0	0	0	4	1	0	0	0	0	0	0	0	0	12
MaRab25	0	0	1	1	0	0	3	1	6	0	0	2	0	4	2	0	0	0	0	0	0	0	0	0	20
MaRab26	1	0	2	1	0	1	0	1	1	0	0	2	0	0	1	0	0	1	1	0	0	0	0	0	12
MaRab27	0	0	0	0	0	1	1	0	2	0	0	0	0	2	2	0	0	0	1	1	0	0	0	0	10
Total	13	1	18	4	7	23	17	44	54	3	7	55	1	80	19	12	8	2	8	7	1	6	10	1	401
