# Supplementary material for: Genome-Wide Analysis of the Rab Gene Family in Melilotus albus Reveals Their Role in Salt Tolerance
Source: Int J Mol Sci. 2022 Dec 21;24(1):126. doi: 10.3390/ijms24010126 (PMC9820615; doi:10.3390/ijms24010126)
Supplement: Supplementary file 1 [file ijms-24-00126-s001.zip › Table S5.pdf]

Table S5. Cis-acting elements in promoters of *MaRab* genes in different group.

Group	auxin-responsive	cell cycle regulation	defense and stress responsiveness	gibberellin-responsiveness	light responsiveness	low-temperature responsiveness	salicylic acid responsiveness	abscisic acid responsiveness	anaerobic induction	auxin responsiveness (MYB binding site)	circadian control	light induction (MYB binding site)	seed-specific regulation	MeJA-responsiveness	zein metabolism regulation	meristem expression	endosperm expression	anoxic specific inducibility	gibberellin-responsive (MYB binding site)	drought-inducibility	flavonoid biosynthetic genes regulation	light induction	MYBHv1 binding site	wound-responsive element	Total
A	3	1	8	1	4	10	10	15	34	0	2	16	0	36	9	4	4	0	3	3	0	3	5	1	172
B	2	0	2	0	1	1	0	2	2	0	2	3	0	4	0	3	0	0	2	0	0	0	0	0	24
C	2	0	2	0	0	4	1	5	7	2	0	9	0	10	3	1	0	1	1	1	0	1	1	0	51
D	2	0	1	1	0	1	0	10	2	0	1	10	0	16	0	1	0	0	0	0	0	0	3	0	48
E	0	0	0	0	0	0	1	0	1	0	0	0	0	2	0	0	1	0	0	0	0	1	0	0	6
F	1	0	0	0	1	3	1	8	2	0	0	11	0	6	0	1	0	0	0	0	0	0	0	0	34
G	1	0	3	0	1	3	4	1	3	1	1	2	0	4	6	2	2	0	1	1	0	0	1	0	37
H	2	0	2	2	0	1	0	3	3	0	1	4	1	2	1	0	1	1	1	2	1	1	0	0	29
Total	13	1	18	4	7	23	17	44	54	3	7	55	1	80	19	12	8	2	8	7	1	6	10	1	401
