# Supplementary material for: Genome-Wide Analysis of the Rab Gene Family in Melilotus albus Reveals Their Role in Salt Tolerance
Source: Int J Mol Sci. 2022 Dec 21;24(1):126. doi: 10.3390/ijms24010126 (PMC9820615; doi:10.3390/ijms24010126)
Supplement: Supplementary file 1 [file ijms-24-00126-s001.zip › Table S6.pdf]

Table S6. Divergence between 13 *MaRab* gene pairs in *M. albus*.

Gene 1	Gene 2	Duplication	*Ka*	*Ks*	*Ka/Ks*
MaRab5 (Malbus0103131.1)	Malbus0304262.1	W	0.091989	0.667796	0.13774988
MaRab3 (Malbus0102734.1)	Malbus0304003.1	W	0.026853	0.652423	0.04115906
MaRab1 (Malbus0100901.1)	Malbus0704541.1	W	0.051028	0.853293	0.0598015
Malbus0100934.1	MaRab26 (Malbus0704571.1)	W	0.152714	0.873756	0.17477927
MaRab8 (Malbus0203104.1)	Malbus0801493.1	W	0.035575	0.460449	0.07726241
Malbus0304064.1	MaRab17 (Malbus0501082.1)	W	0.102754	0.828948	0.12395656
Malbus0302953.1	MaRab16 (Malbus0500112.1)	W	0.032667	0.407585	0.08014805
MaRab11 (Malbus0400281.1)	MaRab22 (Malbus0700190.1)	W	0.022033	0.579968	0.03799072
Malbus0401076.1	MaRab25 (Malbus0703028.1)	W	0.11122	1.092289	0.10182268
MaRab15 (Malbus0404520.1)	Malbus0703473.1	W	0.031001	0.832764	0.03722722
Malbus0503785.1	MaRab19 (Malbus0601485.1)	W	0.042487	0.558984	0.07600804
Malbus0500422.1	MaRab20 (Malbus0603614.1)	W	0.150804	0.817649	0.18443666
MaRab25 (Malbus0703028.1)	Malbus0703473.1	W	0.062163	1.657565	0.03750238

Notes: W, whole genome-wide duplication; Ka, non-synonymous substitution rate; Ks, synonymous substitution rate.
