# Supplementary material for: Genome-Wide Analysis of the Rab Gene Family in Melilotus albus Reveals Their Role in Salt Tolerance
Source: Int J Mol Sci. 2022 Dec 21;24(1):126. doi: 10.3390/ijms24010126 (PMC9820615; doi:10.3390/ijms24010126)
Supplement: Supplementary file 1 [file ijms-24-00126-s001.zip › Table S7.pdf]

Table S7. Databases and websites used in this study.

No.	Soft/Database	Function	Source	Date of Access
1	NCBI database	Obtaining gene sequences and coding sequences (CDSs)	https://www.ncbi.nlm.nih.gov/	20 November 2021
2	Pfam website	Verifing the candidate Rab genes	http://pfam.xfam.org/search/keyword	23 November 2021
3	MEME	Identifing conserved motifs of Rabs	http://meme-suite.org/tools/meme	25 November 2021
4	WebLogo	Creating Rab sequence logos from the multiple alignment sequences	http://weblogo.berkeley.edu/logo.cgi	15 December 2021
5	Swiss-Model website	Predicting 3D structures of Rab proteins	https://swissmodel.expasy.org/	15 January 2022
6	GeneStructure Display Server	Establishing and visualizing all gene structures	http://gsds.cbi.pku.edu.cn/	20 February 2022
7	STRING(v 11.5)	Establishing Protein interaction network	https://cn.string-db.org/	13 June 2022
8	CELLO v.2.5 website	Predicting the subcellular localization	http://cello.life.nctu.edu.tw/	14 June 2022
9	Protsacle tool	Predicting the primary structure of Rab proteins	https://web.expasy.org/protscale	13 December 2021
10	PlantCARE website	Recognizing the cis-acting elements	http://bioinformatics.psb.ugent.be/webtools/plantcare/html	28 December 2021
