# Supplementary material for: Genome-Wide Analysis of the Rab Gene Family in Melilotus albus Reveals Their Role in Salt Tolerance
Source: Int J Mol Sci. 2022 Dec 21;24(1):126. doi: 10.3390/ijms24010126 (PMC9820615; doi:10.3390/ijms24010126)
Supplement: Supplementary file 1 [file ijms-24-00126-s001.zip › Table S8.pdf]

Table S8. Primers used in this study.

Primer name	Sequences
MaRab1_R	GCTGGTTCTCAAGATCAC
MaRab1_F	CCAAGAACGGTATAGAGCA
MaRab5_R	TTACAAGCAACAACCACC
MaRab5_F	GTTGATAGAGAATCTGAAAGGG
MaRab6_R	GCTCTGTACCTTTCTTGAC
MaRab6_F	CGAGGAATCTATTTACCGT
MaRab11_R	CATTAAATCCCTCCTTAGCA
MaRab11_F	CTTATTCAGGCCAGTCCA
MaRab12_R	GCTTCCAAAGCAGAAGTC
MaRab12_F	GTCAGGAAAGGTACCGTG
MaRab13_R	ACTTCAGCAAATGCATTCTC
MaRab13_F	GTCAAGAAAGGTATCGCG
MaRab17_R	CCTCCACATTCTCACCAG
MaRab17_F	CCAAGAAAGGTACAGGGC
MaRab19_R	AATTGTTGCCTGATAGGTG
MaRab19_F	CTTTAGCGAAGTACAAACTG
MaRab22_R	ATCTCTGTTGTCGTCCAC
MaRab22_F	TCTCATTCAGGCTAGTCCA

MaRab23_R	TTTCCCTCTACCTGAAGG
MaRab23_F	TTTGATCGGAGATTCTGG
MaRab25_R	GCACGATATCTTTCTTGTCC
MaRab25_F	GATGACGACTACGATTATCTC
yeast_MaRab1R	tacatgatgcggccctctagaTTACTGACAACACATGCTCTTCT
yeast_MaRab1F	cttggtaccgagctcggatccATGGCGAGTGCAGCAG
yeast_MaRab17R	tacatgatgcggccctctagaTTAACAAGAGCATGAAGACAACTTC
yeast_MaRab17F	cttggtaccgagctcggatccATGGTCCAAGGTGAGAAGAAAC

* Primers names include yeast are required for yeast heterologous expression verification, the rest are required for quantitative real-time PCR.
