# Supplementary figures and images for: Genome-Wide Analysis of the Rab Gene Family in Melilotus albus Reveals Their Role in Salt Tolerance
Source: Int J Mol Sci. 2022 Dec 21;24(1):126. doi: 10.3390/ijms24010126 (PMC9820615; doi:10.3390/ijms24010126)

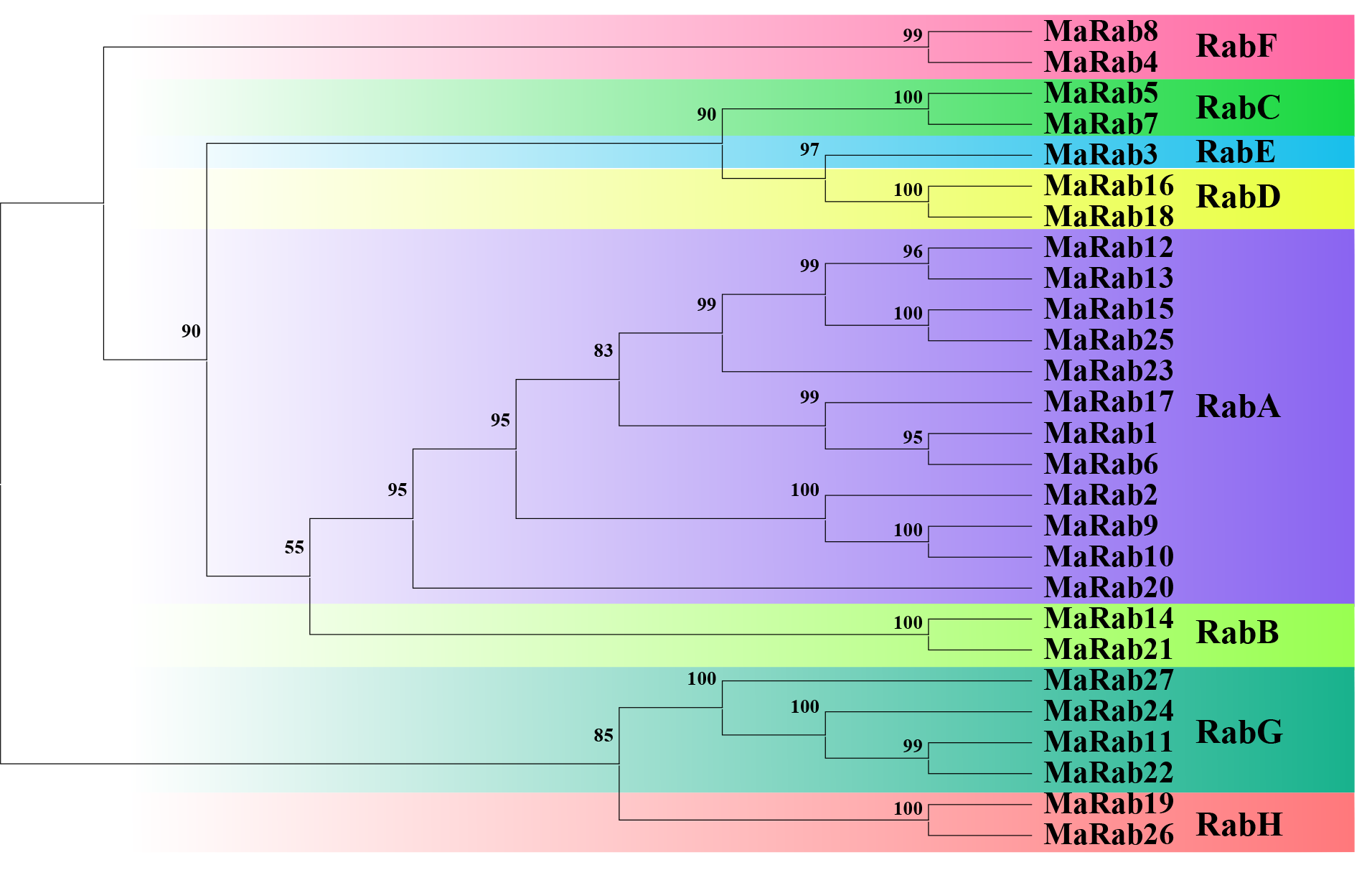

Supplement: Supplementary file 1 [file ijms-24-00126-s001.zip › Figure S1.tif]

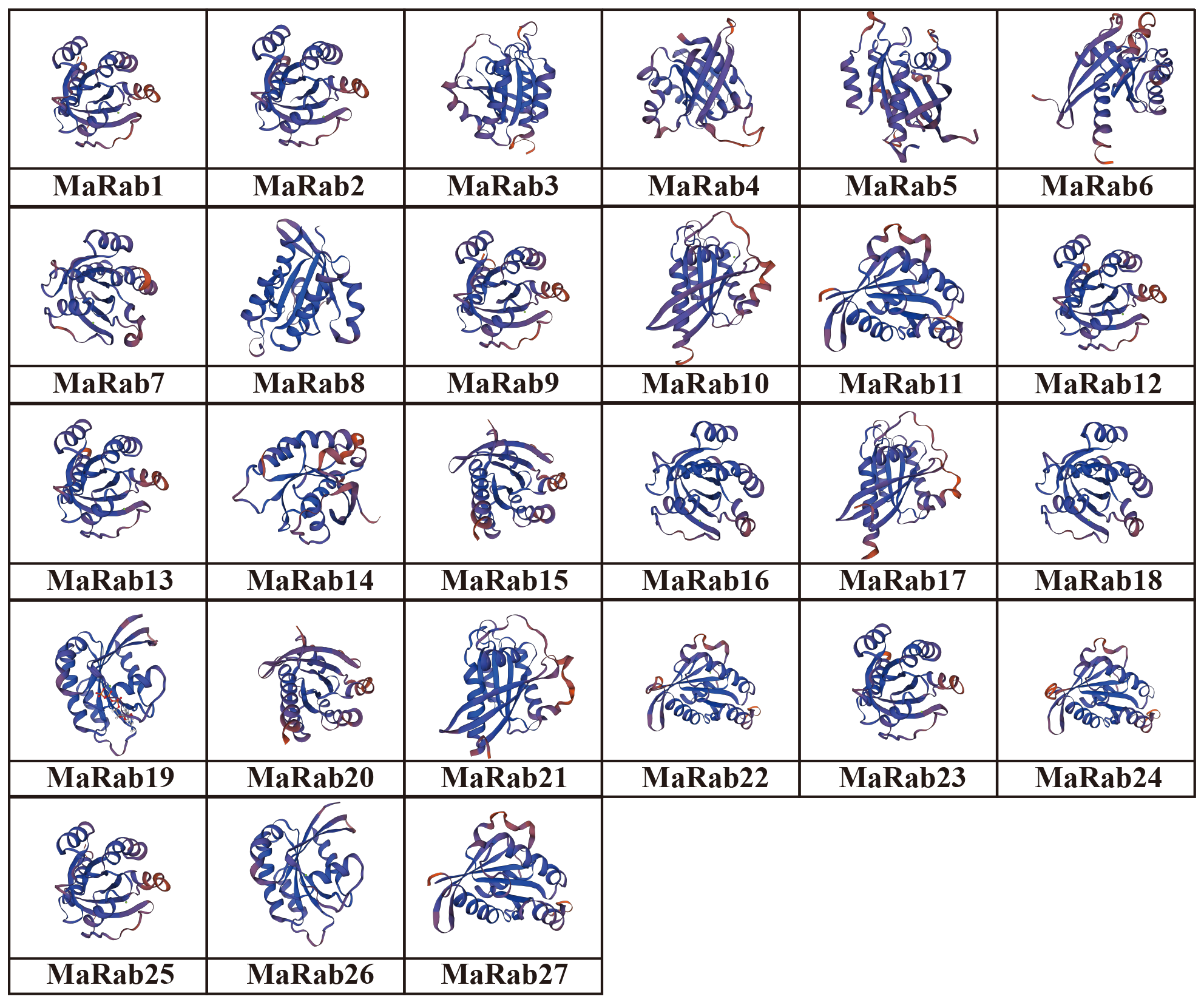

Supplement: Supplementary file 1 [file ijms-24-00126-s001.zip › Figure S2.tif]

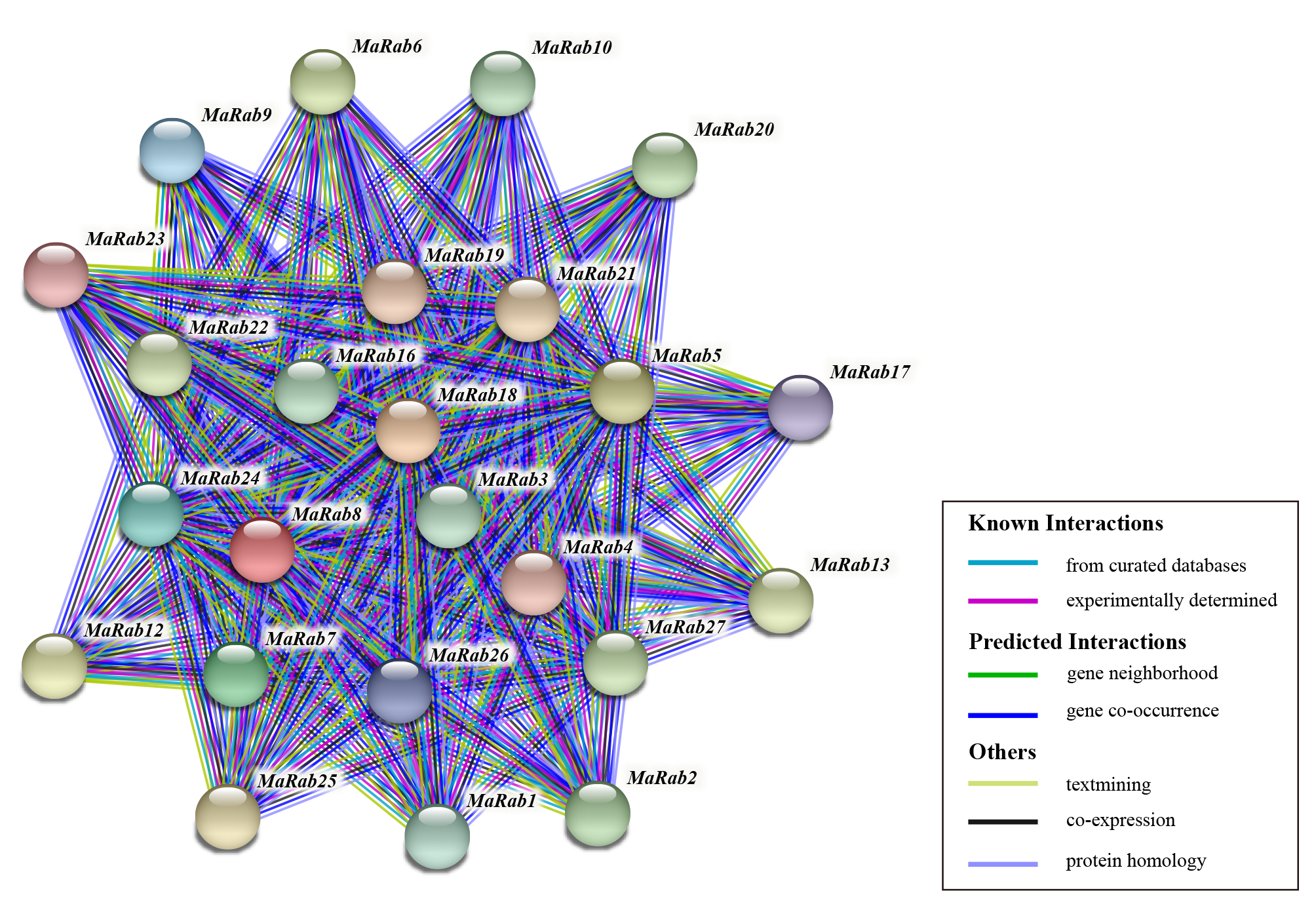

Supplement: Supplementary file 1 [file ijms-24-00126-s001.zip › Figure S3.tif]

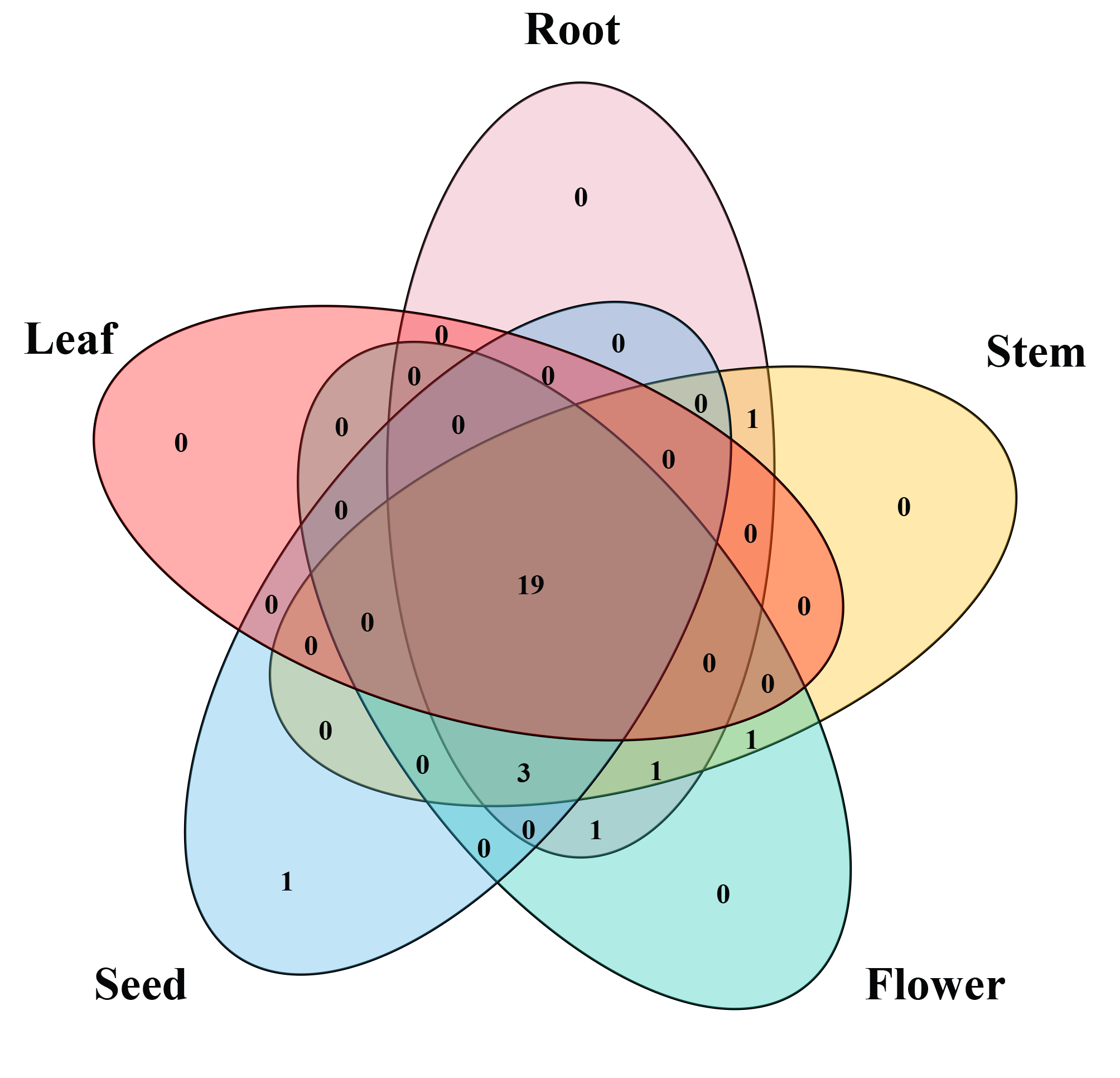

Supplement: Supplementary file 1 [file ijms-24-00126-s001.zip › Figure S4.tif]

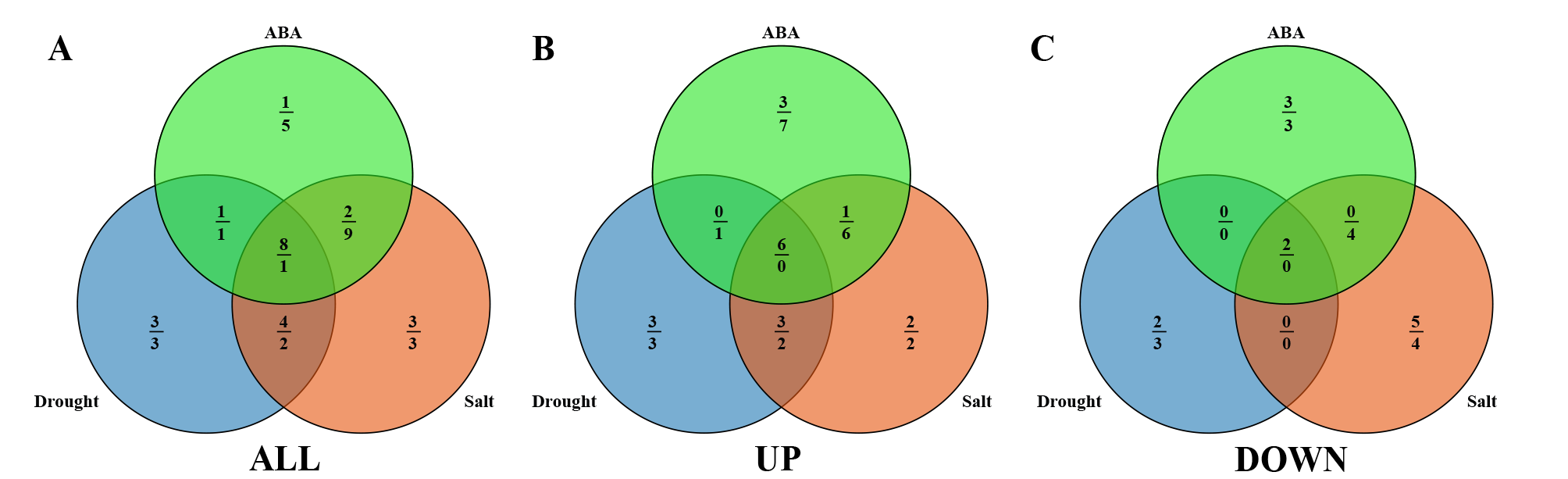

Supplement: Supplementary file 1 [file ijms-24-00126-s001.zip › Figure S5.tif]
